# Endodontic Re‐Surgery With Simultaneous Recession Coverage Using a Modified Flap Design After Failed Primary Apicoectomy: A Case Report

**DOI:** 10.1002/ccr3.71155

**Published:** 2025-10-07

**Authors:** Sohar Flisfisch, Edgar Schäfer, Sebastian Bürklein

**Affiliations:** ^1^ Private Practice Basel Switzerland; ^2^ Department of Operative Dentistry and Periodontology University of Freiburg Freiburg Germany; ^3^ Central Interdisciplinary Ambulance in the School of Dentistry University of Münster Münster Germany

**Keywords:** apicoectomy, bone substitute, calcium silicate cement, surgical technique

## Abstract

Primarily, the present case report shows a coronal shift of the gingival margin after apicoectomy using a new modified coronally advanced flap. Secondly, by using Biodentine, complete bony healing was achieved without any bone substitutes.

## 1 Introduction

Even decades after successful root canal treatment (RCT), an apical periodontitis may develop [1, 2, 3]. Consequently, the need for orthograde retreatment or alternatively a surgical approach should be considered. In cases where the risk of severe tooth damage excludes the option of orthograde retreatment, an apicoectomy is the only minimally invasive option to maintain the tooth [4]. Although high survival rates of apicoectomy are well documented [5], clinicians often face gingival recession in the surgical area [6, 7]. Hence, several flap techniques were developed to address that topic but with sobering outcomes so far [8, 9]. Therefore, a surgical technique based on a recession coverage approach [10] to avoid gingival recession after apical surgery is presented in this case report.

While successful use of calcium silicate cements for retrograde filling is well documented [11], in greater apical lesions, the additional use of bone substitutes can be beneficial [12, 13]. Hence, the present case report shows, furthermore, that smaller apical lesions might resolve without using bone substitutes.

## 2 Case History/Examination

**FIGURE 1:**
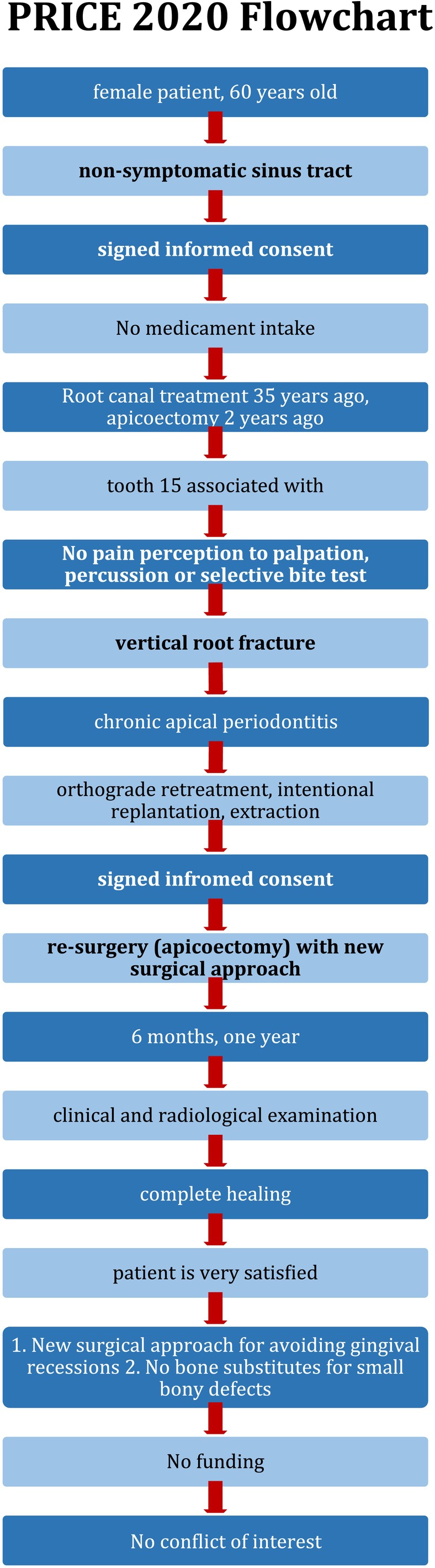
PRICE 2020 flowchart.

The present case report was conducted in accordance with the preferred guidelines for reporting case reports in endodontics (PRICE 2020; Figure 1) [14]. Written informed consent from the patient was obtained.

**FIGURE 2:**
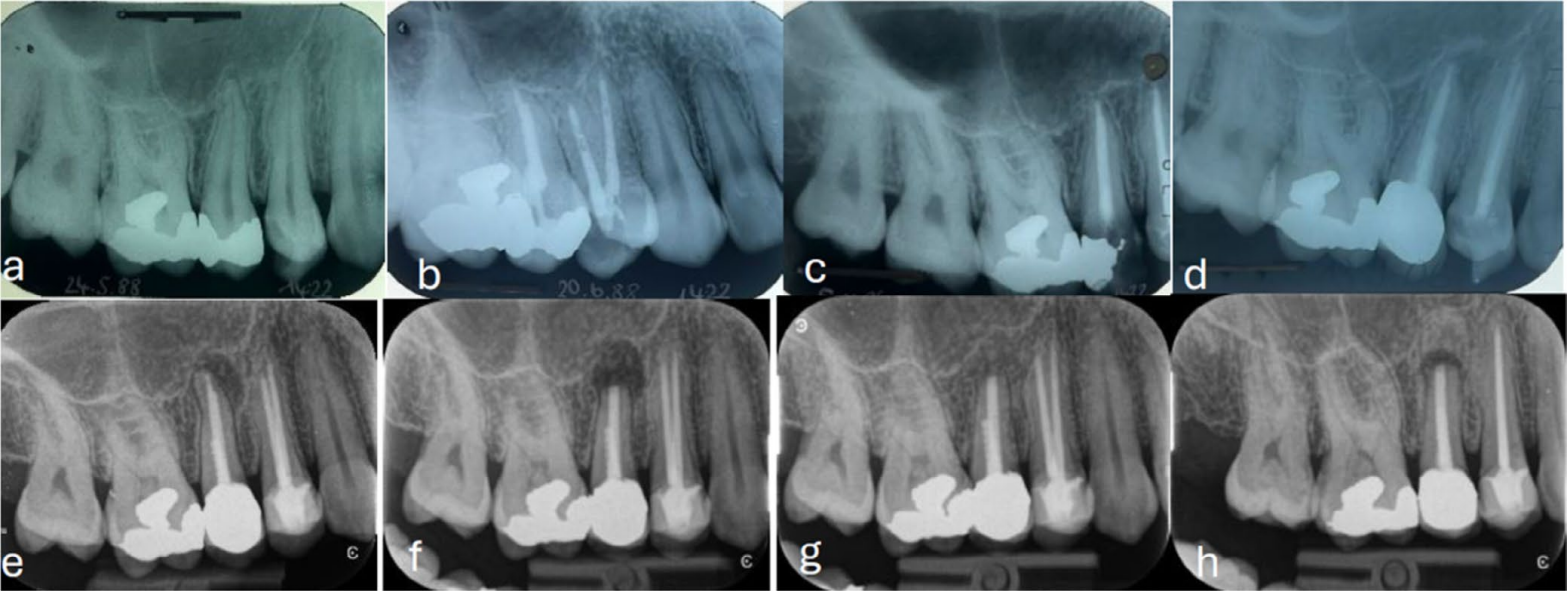
(a) Periapical radiograph (1988) showing a symptomatic apical periodontitis at tooth 15. (b) Root canal filling of teeth 14 and 15 1 month later. (c) Fracture of the palatal wall of tooth 15, 8 years later. (d) Asymptomatic periapical lesion at tooth 15, 18 years after root canal treatment. (e) Symptomatic apical periodontitis at tooth 15, 33 years after root canal treatment. (f) Apicectomy of tooth 15. (g) Radio‐opaque bone filler. (h) Radio‐translucency 1 year after apicoectomy at tooth 15.

In alio loco, the female patient experienced RCT on tooth 15 in 1988 when she was 25 years old due to symptomatic apical periodontitis (Figure 2a,b). Eight years later, the palatal wall of tooth 15 fractured (Figure 2c), and she received a screw retained composite core and consecutively a porcelain‐fused‐to‐metal (PFM) crown. Another 10 years later, a new periapical lesion was evident at tooth 15 (Figure 2d), and the patient was informed about that circumstance, but she was free of symptoms and therefore refrained from any further treatment options. It was another 15 years later, in total 33 years after the initial RCT, when the patient presented herself with tenderness to pressure and chewing pain caused by tooth 15. Clinical examination revealed an exacerbation of asymptomatic apical periodontitis at tooth 15 (Figure 2e). Teeth 14, 15, and 16 presented with gingival recessions type 1 [15]. Due to the intact PFM crown at tooth 15 with an intracanal screw, the risk of root fracture was considered possible when removing the intracanal screw. Hence, the option of orthograde retreatment was discarded in favor of apicoectomy [16]. After root resection and a root‐end filling using MTA (Figure 2f), the remained bony cavity was filled with bone substitute (Figure 2g). Albeit the patient was free of symptoms after the surgical procedure, she reported to have noticed a higher gingival margin with time in the region 14–16.

## 3 Methods—Investigations and Treatment

**FIGURE 3:**
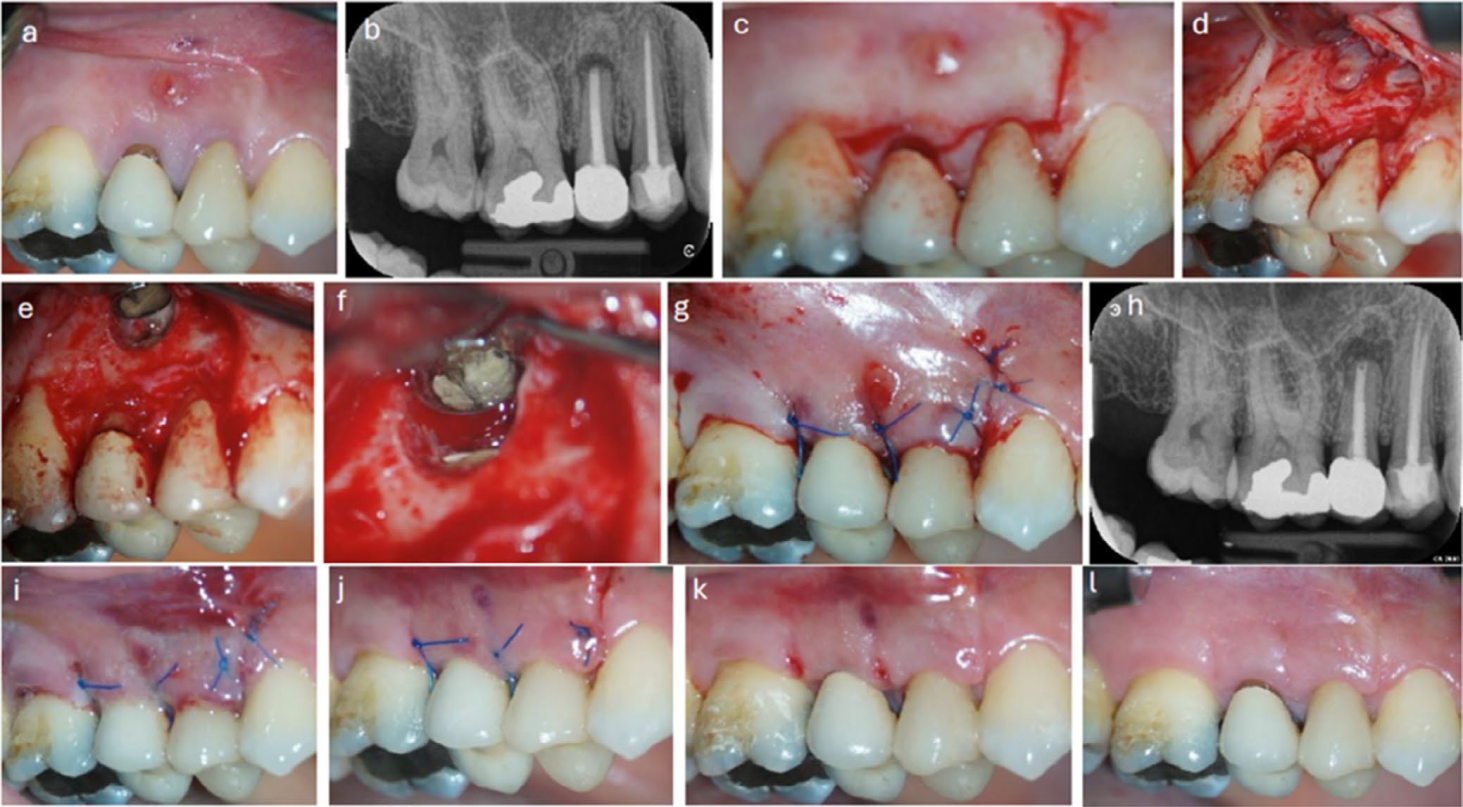
(a) Sinus tract associated with 15. (b) Corresponding radiograph showing a periapical translucency in regio 15. (c) Incision line for coronally advanced flap. (d) Exposition of granulation tissue around former lateral window. (e) Extended resection of the root tip of tooth 15. (f) Apical sealing with Biodentine. (g) Coronally advanced flap with 5/0 monofilament sling sutures. (h) Postoperative radiograph. (i) Clinical situation 1 day after surgery. (j) Clinical situation 7 days after surgery. Removal of interrupted sutures from releasing incision. (k) Clinical situation 11 days after surgery. Removal of sling sutures. (l) Clinical situation 6 weeks after surgery. Almost complete recession coverage of teeth 14, 15 and 16.

Two years later the patient came to our dental office. A sinus tract in the buccal region of tooth 15 was detected while the patient was free of symptoms (Figure 3a). A radiograph revealed a periapical lesion around the resected root of tooth 15 (Figures 2h and 3b). The patient agreed to retreat that area surgically. The surgical procedure was performed under a microscope (Proergo, Zeiss, Oberkochen, Germany). This time a new flap design was applied. A flap technique proposed for the esthetic treatment of multiple recessions [10] was modified, providing sufficient access to the tip of the root. According to the present recessions, surgical papillae were created in split thickness from tooth 14 to tooth 16, as already described [10]. This was followed by intrasulcular incisions, which were connected to previously created surgical papillae. A mesial releasing incision following the scar line from the previous surgical approach was then performed and extended 5 mm beyond the mucogingival line to assure full access with sufficient visibility at the periapical region. From the sulci, the flap was extended apically in full thickness until the periapical region was reached (Figure 3c,d). Anatomical papillae were de‐epithelized, and the flap was released apically of the bony defect from inserting muscles with a deep incision and subsequently with a superficial incision parallel to the oral mucosa, cutting connecting tissue fibers to ensure tension‐free coronal advancement after apical surgery. After exposing the periapical region of tooth 15, granulation tissue was removed, as well as remnants of the bone substitute, and the resected root was slightly more resected with a sterile diamond drill. A new root‐end cavity was piezo surgically (E10D tip, EMS, Nyon, Switzerland) prepared (Figure 3e). Consecutively, the root‐end cavity was sealed with Biodentine (Septodont, Niederkassel, Germany) with no additional biomaterial used for the bony lesion (Figure 3f). The flap was then coronally advanced, moving the created surgical papillae onto the de‐epithelized anatomical papillae and stabilized with 5/0 monofil (Seralon, DSS‐18, Serag Wiessner, Naila, Germany) sling sutures [17]. Single oblique interrupted sutures were used for the releasing incision to secure the coronal advancement (Figure 3g), and a radiograph was taken (Figure 3h). Ten days later, after uneventful healing, the sutures were removed (Figure 3i–k). Six weeks later, healthy soft tissue with almost complete root coverage of tooth 14–16 was present (Figure 3l). All clinical photographs were taken with either a Sony α7III or Sony α NEX‐5t (Sony, Tokyo, Japan). Neither the photographs nor the radiographs were adjusted regarding image parameters. All radiographs were viewed by the same operator (S.F.) under identical conditions using the same monitor. Periodontal measurements were recorded before and 1 year after surgery (Table 1).

**TABLE 1:** Periodontal measurements of teeth 14, 15 and 16 before and after endodontic re‐surgery.

Tooth	Periodontal measurements before re‐surgery												Periodontal measurements after re‐surgery											
	PPD			GR			CAL			BOP			PPD			GR			CAL			BOP		
	mb	b	db	mb	b	db	mb	b	db	mb	b	db	mb	b	db	mb	b	db	mb	b	db	mb	b	db
	mp	p	dp	mp	p	dp	mp	p	dp	mp	p	dp	mp	p	dp	mp	p	dp	mp	p	dp	mp	p	dp
14	3	2	3	0	−2	0	3	4	3	−	−	−	3	2	3	0	−0.5	0	3	2	3	−	−	−
	3	2	2	0	0	0	3	2	2	−	−	−	3	2	3	0	0	0	3	2	3	−	−	−
15	3	2	3	0	−2	0	3	4	3	−	+	+	3	2	3	0	0	0	3	2	3	−	−	−
	3	2	3	0	0	0	3	2	3	−	−	−	2	2	2	0	0	0	2	2	2	−	−	−
16	2	1	2	0	−3	0	2	4	2	−	−	−	2	2	2	0	0	0	2	2	2	−	−	−
	3	1	2	0	0	0	3	1	2	−	−	−	2	2	2	0	0	0	2	2	2	−	−	−

*Note:* In blue buccal gingival recessions before and after endodontic re‐surgery.

Abbreviations: BOP, bleeding on probing; CAL, clinical attachment loss; GR, gingival recession; PPD, probing pocket depth.

## 4 Conclusions and Results

**FIGURE 4:**
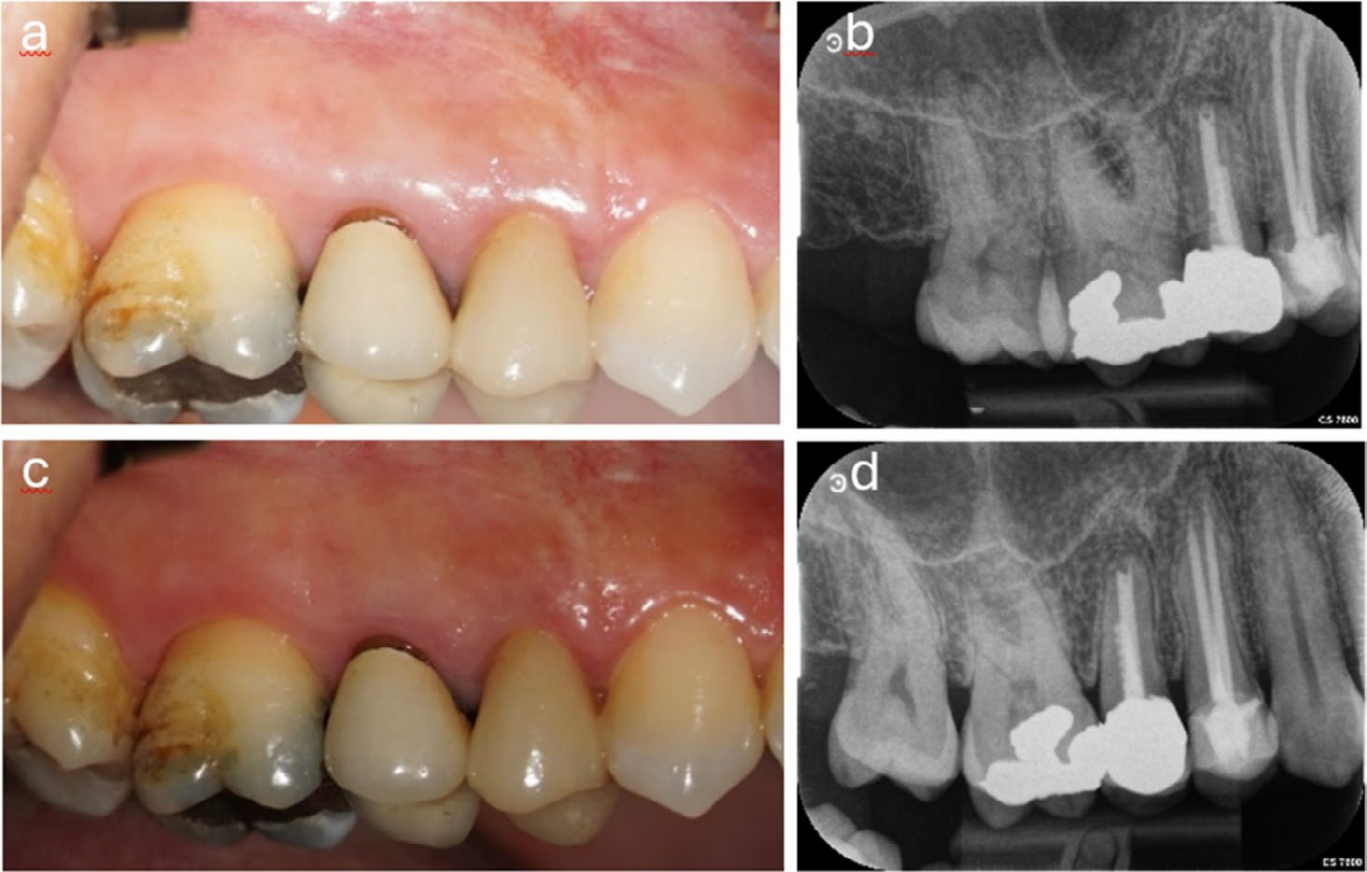
(a, b) Healing 8 months after surgery with corresponding radiograph. (c, d) Healing 14 months after surgery with corresponding radiograph showing periapical radiopacity.

Eight and 14 months later, instead of further gingival recession at tooth 15 an almost complete root coverage including the adjacent teeth could be preserved, which considerably enhanced patient's satisfaction. Additionally, a radiological opacity was observed around the periapical region of tooth 15, which can be interpreted as bony healing (Figure 4a–d). A coronally advanced flap with a single releasing incision after apicoectomy is described, which can prevent and even improve gingival recessions with a minimal risk of scar tissue formation. This is because the beforehand created surgical papillae covered perfectly the de‐epithelized anatomical papillae when advancing the entire flap coronally. While probing pocket depth remained stable, gingival recession and consequently clinical attachment level improved significantly even of the adjacent teeth (Table 1). With the use of Biodentine as apical sealant bone substitutes could be neglectable.

## 5 Discussion

The present case report shows the history of tooth 15 that developed an exacerbation of an apical periodontitis 33 years after RCT. Etiologically, the asymptomatic apical periodontitis might be the consequence of the fractured palatal wall 8 years after RCT, with the possibility of contamination of the existing root canal filling. 25 years after the final prosthetic restoration, the patient developed a symptomatic apical periodontitis, which was addressed by apicoectomy using MTA as retrograde filling material and with a bone substitute to fill the bony defect.

The first surgical approach must have been performed with the papilla base incision flap that can be assumed based on the scar formation. This flap was developed to avoid gingival recession after apical surgery [18]. The outcome was a deterioration of the gingival recession in comparison to baseline according to patient perception. This is in accordance with reports in the literature comparing papilla base incision flap with the sulcular incision technique [19]. In both surgical approaches, gingival recessions were observed equally. Submarginal incision techniques provide the best results for gingival recession, keeping the gingival margin on the level from baseline [20]. However, scar tissue formation is mostly pronounced using latter techniques [21]. To overcome these issues, a new surgical technique is described in this case report, which was applied for the necessary re‐surgery. A coronal advancement of the flap is described to even cover a preexisting gingival recession—not simply aiming at the avoidance of recession progression. With only one mesial releasing incision, scar tissue formation is considerably reduced. The patient in this present case had a thick phenotype. A thin phenotype is considered to create more gingival recessions, applying conservative techniques [22]. This could be overcome by combining the presented new technique with connective tissue graft to transform a thin phenotype into a thick phenotype [23]. Albeit, recently, the influence of the gingival phenotype on root coverage procedures has been questioned as aesthetic results were not compromised by its initial classification [24]. Thus, the proposed technique may also be applicable for thinner phenotypes. However, to provide a stable gingival margin in the long term, a thick phenotype seems to be superior [25]. Although coronally positioned flap techniques have already been used in periodontal treatment to avoid or correct gingival recessions, this is the first time that this technique is presented for its use in endodontics.

The positive biological effect of calcium silicate cements on osteoblasts is well known [26]. However, the biocompatibility of MTA and Biodentine is similar [27]; the sealing ability of Biodentine was reported to be superior to MTA [28, 29, 30]. This difference between MTA and Biodentine might be a potential cause of failure of the first surgery.

Furthermore, the use of biomaterials for enhanced healing of periapical lesions is well documented [31, 32]. However, most studies described these positive effects for lesions with diameters greater than 10 mm. In the present case, the diameter of the periapical lesion was approximately 8 mm. This might result in a higher density of the bone substitute resulting in a stronger contact to the calcium silicate cement sealing. Hence, it might be speculated that this could negatively influence the resistance of the cement, which in turn might have resulted in apical leakage and failure of the first surgical intervention [33]. In addition, the radiological pattern of bone substitutes with their similarity to the original tissue can hamper the differentiation between grafts and periapical disease [34].

The fact that a CBCT scan was not used before the second surgery might be regarded as a certain limitation. However, as no indications of an atypical anatomy or other reasons explaining the apical pathology were present, the ALARA principle of radiation safety was taken into account.

## Author Contributions

**Sohar Flisfisch:** writing – original draft. **Edgar Schäfer:** writing – original draft. **Sebastian Bürklein:** writing – original draft.

## Consent

Written informed consent was obtained from the patient to publish the present case report in accordance with the journal's patient consent policy.

## Conflicts of Interest

The authors declare no conflicts of interest.

## Data Availability

Data available on request from the authors.
